# Identification of the Maize Gravitropism Gene *lazy plant1* by a Transposon-Tagging Genome Resequencing Strategy

**DOI:** 10.1371/journal.pone.0087053

**Published:** 2014-01-31

**Authors:** Thomas P. Howard, Andrew P. Hayward, Anthony Tordillos, Christopher Fragoso, Maria A. Moreno, Joe Tohme, Albert P. Kausch, John P. Mottinger, Stephen L. Dellaporta

**Affiliations:** 1 Department of Molecular, Cellular and Developmental Biology, Yale University, New Haven, Connecticut, United States of America; 2 International Center for Tropical Agriculture, Cali, Colombia; 3 Department of Cell and Molecular Biology, University of Rhode Island, Kingston, Rhode Island, United States of America; University of Guelph, Canada

## Abstract

Since their initial discovery, transposons have been widely used as mutagens for forward and reverse genetic screens in a range of organisms. The problems of high copy number and sequence divergence among related transposons have often limited the efficiency at which tagged genes can be identified. A method was developed to identity the locations of *Mutator (Mu)* transposons in the *Zea mays* genome using a simple enrichment method combined with genome resequencing to identify transposon junction fragments. The sequencing library was prepared from genomic DNA by digesting with a restriction enzyme that cuts within a perfectly conserved motif of the *Mu* terminal inverted repeats (TIR). Paired-end reads containing *Mu* TIR sequences were computationally identified and chromosomal sequences flanking the transposon were mapped to the maize reference genome. This method has been used to identify *Mu* insertions in a number of alleles and to isolate the previously unidentified *lazy plant1 (la1)* gene. The *la1* gene is required for the negatively gravitropic response of shoots and mutant plants lack the ability to sense gravity. Using bioinformatic and fluorescence microscopy approaches, we show that the *la1* gene encodes a cell membrane and nuclear localized protein. Our Mu-Taq method is readily adaptable to identify the genomic locations of any insertion of a known sequence in any organism using any sequencing platform.

## Introduction

The maize *Mutator* (*Mu*) is one of the most aggressively mobile transposon families yet characterized in any organism. *Mutator* lines were first described as a genetic system that increased the mutation rate by 30-fold [1]. The “Mutator” trait did not segregate according to a simple one-gene model, with nearly 100% of progeny between crosses of *Mutator* lines and non-*Mutator* lines exhibiting high mutation rates [1]. The first mutant allele to be cloned and characterized from a *Mutator* line contained a 1.4 kb insertion (later named *Mu1*) in the alcohol dehydrogenase (*adh1*) gene of maize [2]. The *Mu1* element had ∼215 bp highly homologous terminal inverted repeats (TIRs), and was flanked by a 9 bp target sequence duplication at its site of insertion [3]. When Robertson’s *Mu* lines were examined by Southern hybridization, plants were shown to possess between 10–70 copies of *Mu*-related sequences [4]. These early studies suggested that active transposable elements were the genetic basis for the high mutation rates found in Robertson’s *Mutator* lines.

Continued characterization of *Mu* lines has elucidated a family of transposons that is both diverse and complex. More than a dozen different *Mu* elements are currently known (reviewed in [5]), including the autonomous *MuDR* element that encodes for an active transposase, MURA [6], [7]. All active (mobile) *Mu* elements share highly similar TIRs but their internal regions can vary considerably in both size and sequence [reviewed in [5], [8]–[11]]. MURA binds to a highly conserved region within the *Mu* TIR, promoting transposition both in *cis* and in *trans* for other *Mu* elements found in the plant genome [7]. In this way, the presence and activity of *MuDR* determines whether all non-autonomous *Mu* elements are mobile or immobile in the plant’s genome. Other epigenetic factors, such as the cytosine methylation state of *Mu* elements, affect mobility of individual elements (reviewed in [5]).

A number of factors account for the exceptionally high mutation rates in *Mu* lines. First, active *Mu* lines can contain over 100 *Mu* elements per genome (reviewed in [9]), a number that can be maintained from generation to generation [12]. On average, each transposon is responsible for one new insertion event every generation, either non-conservatively through transposition or conservatively through duplication [13]. Studies show that *Mu* elements prefer to insert into low copy number (non-repetitive) DNA [14], and genome-wide analysis suggests that *Mu* elements preferentially insert into regions of the genome that are transcribed [15]. For instance, analysis of the *RescueMu* element (described below) shows that 69% of its insertion sites are located in putatively expressed genomic sequences [16] even though the maize genome appears to be made up of more than 80% repetitive sequences [17]. Finally, *Mu* elements exhibit little target site sequence specificity, allowing for essentially uniform genome-wide mutagenesis (reviewed in [9]). These studies show that *Mu* elements are abundant, highly active, and targeted towards genes throughout the entire genome, features that drive a forward mutation rate of up to 10^−3^ per locus per generation.

Given their robust mutagenic properties, *Mu* transposons have been employed in a number of gene cloning and functional genomics experiments. Originally, mutant alleles were recovered from *Mu* lines, and the corresponding gene cloned by their association with *Mu* sequences by standard transposon tagging methods [18]–[20]. The limitation with this approach was the high copy number and diversity of *Mu* elements, making the linkage association of any particular element with a mutant allele a difficult and time-consuming endeavor. In fact, using this approach, only a handful of genes had been cloned even decades after the molecular identification of *Mu* elements.

More efficient reverse genetic screens became feasible with the advent of PCR methods that incorporated a degenerate *Mu* primer that anneals to a TIR consensus sequence together with a gene-specific primer to clone *Mu*-induced mutant alleles [20], [21]. The Maize-Targeted-Mutagenesis (MTM) project increased the scope of reverse genetic screens by producing a large population (>40,000) of *Mu* plants whose genomic DNA was multiplexed for screening for *Mu* insertions in specific gene sequences [22]. Since these methods required prior knowledge of a gene sequence for primer design, they did not necessarily provide a robust approach for forward genetic studies of *Mu*-induced mutant phenotypes.

Other approaches were developed to clone and sequence *Mu* flanking sequences (MFS) *en masse* without prior knowledge of the gene surrounding the insertion [23]. These “anonymous” methods would ligate adapters to restriction-digested genomic DNA, and then amplify DNA with a degenerate *Mu* TIR primer together with a primer specific to the adapter sequence. A variation of this method, called MuTAIL-PCR, was developed to achieve the same goal with fewer sample manipulations, incorporating nested degenerate *Mu* TIR primers together with random primers and programmed thermal alternations to gradually enrich for MFS [24]. With these technologies, numerous groups undertook large-scale MFS cloning and sequencing projects [15]. Chief among these was the “UniformMu” project, which generated extensive maize *Mu* populations and incorporated the ability to stabilize transpositions by epigenetically silencing *Mu* elements after a cycle of mutagenesis [25]. UniformMu studies identified almost 2,000 independent, stable insertions, creating, at the time, one of the most significant knockout resources in maize [26]. While these resources provide efficient reverse genetics methods of identifying insertions in known genes, they have not been widely employed as a method of conducting forward genetic screens.

The *Rescue Mutator* (*RescueMu*) project attempted to develop a facile transposon tagging strategy by introducing a recombinant *Mu* element containing a bacterial origin of replication (*ori*) and an antibiotic resistance gene (ampicillin) into maize harboring *MuDR* as a transposase source [16], [27], [28]. After rounds of transposition, genomic DNA was digested around *RescueMu*, the linear fragments were circularized by ligation, rescued in *E. coli,* and sequenced to identify the MFS. The main limitation of this method is that all *Mu* elements, including endogenous ones, are active in a *MuDR* line and any one could have caused a mutation of interest. Because of this, only a small subset of mutants derived from *RescueMu* lines was caused by the insertion of *RescueMu* and many mutations went uncharacterized at a molecular level. Nevertheless, this project, like MTM and UniformMu, contributed to the genetic resources in maize by providing an additional source of *Mu*-derived alleles.

The advent of next-generation sequencing (NGS) technologies has increased the throughput and efficiency of *Mutator* functional genomics. The UniformMu project now incorporates Illumina sequencing strategies with over 12,000 of the nearly 40,000 maize genes identified as *Mu*-tagged with 20,000 new insertions expected each year [29]. A recent method called Digestion-Ligation-Amplification (DLA) combines an adapter-ligation step together with NGS to efficiently identify *Mu* flanking genomic sequences [30]. More specifically, DLA employs a degenerate set of *Mu* TIR primers together with a single-stranded, blocked adapter to selectively amplify MFS fragments. A secondary PCR step is required to create a suitable fragment library for sequencing. The DLA method generates a large number of sequencing reads with 94% corresponding to MFS with exceptional depth of coverage. The method was used to identify 12/14 *Mu-*induced alleles of the *glossy4* gene [30]. An alternate NGS-based method, employing a biotinylated oligonucleotide corresponding to the *Mu* TIR, enriches for MFS reads by hybridization and selection from whole genome Illumina libraries [31]. This method has been shown to have an 80% success rate in identifying *Mu*-tagged alleles.

Both of these NGS-based methods are effective at characterizing *Mu* insertion sites, but require specific primers and multiple PCR steps. Here, we report on a method that is both efficient and cost-effective in identifying MFS using a standard sequencing library protocol with minor modifications. Our “Mu-Taq” method incorporates restriction-digested genomic DNA with standard Illumina library protocols for paired-end sequencing to enrich for MFS-containing reads. MFS reads were identified *in silico* with custom scripts and mapped to the maize reference genome to locate *Mu* insertion sites.

Using the Mu-Taq method, we identified several known *Mu-*induced mutations and isolated the previously uncharacterized gravitropism *lazy plant1* (*la1*) gene of maize. To confirm our identification of *la1*, we characterized a second, independent *la1* mutation arising from insertion of a TIR family CACTA transposon in a plant displaying the *lazy* phenotype. We further characterized the wildtype *La1* gene using bioinformatic and fluorescence microscopy approaches. The maize LA1 protein was found to localize to the cell membrane and nucleus, with nuclear localization dependent upon a predicted nuclear localization sequence (NLS).

## Materials and Methods

### Genetic Stocks

The sources of mutant alleles used in this study are listed in Table S1. Mutant germplasm was obtained from the Maize Genetics Cooperation Stock Center (http://maizecoop.cropsci.uiuc.edu/). Many of these alleles were first identified during D.S. Robertson’s original *Mu* experiments and, in some cases, the molecular basis of the mutation was unknown. The “lazy plant” mutation arose as a segregating recessive mutation in an F2 family derived from *Mutator* lines grown at the University of Rhode Island Experiment Station (this study). This mutation was phenotypically similar to the *lazy plant1* mutation first described in 1931 [32] and later designated *la1-mu1*.

### Library Construction

Genomic DNA was isolated as previously described [33]. Approximately 5 µg of DNA was digested by 30 units *Taq* ^α^I restriction endonuclease (New England BioLabs, Inc.) according to the manufacturer’s instructions. The enzyme was inactivated by phenol:chloroform (1∶1) extraction, precipitated with two volumes of ethanol, and resuspended in 20 µl 10 mM Tris pH 8.0. *Taq* ^α^I digestion leaves a 5′ overhang on the ends of fragments. One µg of *Taq* ^α^I digested DNA was made blunt using the NEBNext End Repair Module (NEB) according to the manufacturer’s instructions. Reactions were incubated at 20° for 30 min followed by 65° for 20 min. The reaction buffer was exchanged with 10 mM Tris pH 8.0 in a volume (∼30 µl).

The concentrated end-repaired fragments were 3′-adenylated in a 50 µl reaction using the NEBNext dA-Tailing Module (NEB) according to the manufacturer’s instructions. Reactions were incubated at 37° for 30 min followed by 65° for 20 min. The reaction buffer was exchanged with 10 mM Tris pH 8.0 and the adenylated fragments were ligated to Illumina paired-end adapters (Table S2) using the following conditions: 19.6 µl of adenylated fragments in a 30 µl reaction containing adapters (final concentration 0.33 µM), 3,000 units T4 DNA Ligase (NEB), and 1X T4 DNA Ligase Reaction Buffer (NEB) supplemented with 2 mM ATP (NEB). Reactions were incubated at 20° for 60 min followed by 65° for 15 min. Unligated adapters were removed using the QIAquick PCR Purification Kit (Qiagen) according to the manufacturer’s protocol. Samples were eluted in 30 µl elution buffer prewarmed to 65°.

The adapted fragments were amplified by PCR using modified Illumina PCR primers (Table S3). Triplicate 50 µl reactions were performed for each allele. Reactions contained 1X Phusion High-Fidelity PCR Master Mix with GC Buffer (NEB) or 1X KAPA HiFi HotStart ReadyMix (Kapa Biosystems) (preferred), 60 nM of each primer, and ∼50 ng adapted DNA. Cycling instructions were as follows: for Phusion: 98° (2 min); 12 cycles of 98° (10 sec), 65° (30 sec), 72° (30 sec); 72° (5 min); for KAPA: 95° (4 min); 12 cycles of 98° (20 sec), 65° (15 sec), 72° (15 sec); 72° (5 min). Triplicate samples were pooled and concentrated using Microcon YM-30 Centrifugal Filter Devices (Millipore) as described. Pooled samples were subjected to gel electrophoresis and 200–800 bp fragments were excised and purified using Freeze ‘N Squeeze DNA Gel Extraction Spin Columns (Bio-Rad) according to the manufacturer’s protocol. The samples’ buffer was exchanged to 1X TE and concentrated to a minimal volume.

### Barcoded Adapter Design and Preparation

In these experiments, custom bar-coded Illumina adapters were employed for library preparation. Custom adapters incorporated a unique four-base “barcode” sequence (see Table S2 for adapter sequences). The barcode sequence was sequenced in the first four nucleotides in each of the paired-end reads followed by the thymine added in the adenylation reaction. Note, however, that our method incorporates ligating adapters to adenylated library fragments. Therefore, standard Illumina paired-end adapters, with or without indexing, can be substituted in lieu of custom barcoded primers without any modifications to the protocol.

### Multiplexing and qPCR Library Normalization

The gel-extracted libraries were subjected to qPCR analysis in order to normalize the amount of DNA from each library in the multiplexed sample. The qPCR reaction employed primers (Table S3) that anneal to the extension of the adapters that was added to fragments during the PCR amplification step. The qPCR step estimates the relative concentration of only those fragments with suitable adapters at both ends for sequencing. Triplicate 20 µl reactions were performed containing 1X KAPA SYBR FAST Universal qPCR Master Mix (Kapa Biosystems), 200 nM of each primer, and 1 µl (<1 ng) DNA. Cycling conditions were as follows: 95° (3 min); 40 cycles of 95° (3 sec), 60° (30 sec); ramp to 95° over 5 min; in an Eppendorf Mastercycler ep realplex real-time PCR system, with Ct values calculated using the CalQPlex algorithm. Relative concentrations of each sample were calculated from the mean of triplicate Ct values using a ΔCt method (2^Ct1 − Ct2^). Samples were multiplexed according to their calculated relative concentrations. The multiplexed pool was then concentrated to a minimal volume using the Microcon columns as described. The pool was paired-end sequenced (2×75 nt) using an Illumina HiSeq 2000 at the W.M. Keck Foundation’s Yale Center for Genome Analysis (YCGA).

### Data Analysis

Raw image files were processed by the YCGA using the Illumina CASAVA computational pipeline for base calling and quality score determination. The paired-end fastq reads were demultiplexed and parsed into separate fastq files according to the four-bp terminal barcode using a custom Java script. This algorithm also removed the first five bases of the read, which represents the barcode along with the thymine introduced in the adenylation and adapter ligation steps. In some cases, the parsed fastq files were mapped against the reference maize genome using Burrows-Wheeler algorithm [34]. The parsed fastq files were then analyzed using a second custom script, which identified and parsed reads that contain the *Mu* TIR tag on one or the other end (read 1 or read 2). The script also extracted the sequences of each MFS (31 bases adjacent to the *Mu* TIR tag) into a separate fasta sequence file. The MFS were mapped using the BLASTn algorithm [35] against the *Zea mays* B73 reference genome v2 (maizesequence.org) [17]. BLAST output files were imported into Microsoft Excel for all subsequent analyses. All primary sequence files, extracted MFS, and scripts are provided in File S1.

### Localization Studies

#### Fusion constructs

Plasmid pYU2973 (*La1*:Citrine) was derived from plant expression vector pPZP200b and contained the full-length *La1* CDS with the stop codon removed in frame with the citrine CDS under control of the single CaMV 35S promoter with TEV leader and the 35S terminator (construction details available upon request). Plasmid pYU2972 (La1ΔNLS:Citrine) contained *La1* CDS with an in-frame deletion of 381 bases encompassing amino acids 263–389 of the LA1 protein, and was otherwise identical to pYU2973. The La1ΔNLS:Citrine deletion arose from spontaneous PCR amplification error. The single CaMV 35S promoter was amplified from plasmid pDPG165. The TEV leader sequence and 35S terminator were amplified from plasmid pRTL2.

#### Transient protein expression and confocal microscopy

*Agrobacterium-*mediated transient expression was performed in *N. benthamiana* leaves as previously described [36]. Briefly, *Agrobacterium* GV2260 containing pYU2972 or pYU2973 was grown overnight, pelleted, and resuspended in infiltration medium containing 10 mM MgCl2, 10 mM 2-Morpholinoethanesulfonic acid and 200 µM acetosyringone. Strains were induced at room temperature for 4 hours followed by vacuum infiltration into 4–5 week old *N. benthamiana* leaves at OD_600_ 1.2. Live tissue imaging was performed on a Zeiss LSM510 META confocal microscope (Carl Zeiss) using a 40× C-Apochromat water immersion objective lens. Tissue samples were cut from *N. benthamiana* leaves at approximately 42 hours post infiltration. The 514 nm laser line of an argon laser with appropriate emission filters was used to image citrine and chloroplasts.

## Results

### Identification of a Conserved Motif in *Mu* Elements

We sought to devise a simple and efficient NGS-based method for identifying transposon-induced mutations by virtue of their association with a conserved terminal inverted repeat (TIR) sequence. Current enrichment methods for transposon sequences suffer from limitations, such as complex and costly library preparation, the use of degenerate primers, or assumptions about sequence conservation in *Mu* TIRs. Often, these methods are designed for a single transposon family, or a single sequencing platform, or both, and are not flexible and adaptable.

To address these limitations, we tested a simple transposon enrichment method based on digestion of genomic DNA with a restriction enzyme that recognizes a conserved sequence motif in its TIR element. The enrichment strategy was to digest genomic DNA with an appropriate restriction enzyme as to define one end of the genomic fragments in the TIR element. Given the high read coverage of current instrumentation –200 million or more paired-end reads – these TIR-containing fragments could be readily identified by computational methods and mapped to a reference genome.

In this study, the transposon of interest was Robertson's *Mutator*, a diverse family of transposable elements in maize that share conserved TIRs [reviewed in [5]]. Illumina libraries were constructed using restriction-digested genomic DNA. The choice of the restriction enzyme was crucial since it must recognize a highly conserved or invariant motif in all TIRs found in active *Mu* elements to enrich for these fragments and to maximize the chances that all, or nearly all, *Mu* Flanking Sequences (MFS) were represented in the library. Furthermore, the restriction site needed to be an optimal distance from the end of the TIR to enable identification of TIRs and to map flanking sequences. In the Illumina sequencing protocol, for instance, only 75–100 nt of sequence are generated from one or both ends of library fragments [37]. Therefore, if one end of the library fragment is fixed within the TIR, then the chosen site must be located so as to generate sufficient TIR sequence (*Mu* tag) for identification, as well as sufficient flanking genomic sequence (MFS) for mapping to a reference genomic location. Paired-end read technology was chosen so that either read of a pair could be examined for a TIR tag. Moreover, if necessary, the paired, non-TIR end could be later used for resolution or confirmation of the mapping location of any individual MFS.

An additional requirement is that the restriction site be frequent enough to generate fragment sizes appropriate for NGS methods. For instance, Illumina library fragments are typically between 200 and 800 bp [38]. An enzyme with a recognition site of *N* bp will cut once every 4*^N^* bp [excluding degenerate recognition and assuming 50% G-C genome composition]. In our protocol, the library preparation steps add an additional 130 bp of adapter sequences to every fragment. For example, an enzyme with a 4 bp non-degenerate recognition site would generate a library consisting of fragments normally distributed around 386 bp, an ideal size for Illumina-based sequencing methods. Lastly, it would be preferable that the enzyme not be inhibited by CG or CNG methylation since plant genomes contain high levels of cytosine methylation at these sites [39], [40]. Hence, a methylation-sensitive enzyme will be blocked from digesting a significant fraction of its recognition sites that are methylated, increasing the average fragment size in the library. Taken together, we sought to identify a cytosine methylation-insensitive restriction enzyme that recognizes a conserved 4 bp motif located approximately 20–50 bp from the end of *Mu* TIRs. Depending on its exact location, Illumina paired-end sequencing (2×75 nt) would yield a suitable 20–50 nt TIR tag for fragment identification and a suitable 20–50 nt of MFS for reference mapping.

**Figure 1:**
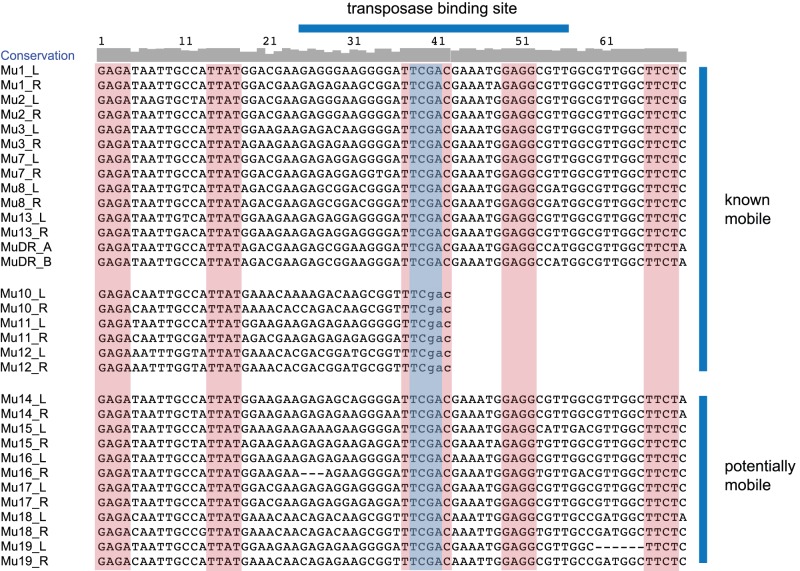
Alignment of *Mu* Terminal Inverted Repeats (TIRs). ClustalW was used to align all known active and potentially active *Mu* elements. Strings of four or more consecutive bases that are entirely conserved among all elements are shaded in red. The conserved *Taq* ^α^I site is shaded in blue. *Mu4, 5,* and *6* are inactive and are not included [reviewed in [5]]. *Mu9* is MuDR. Only the first 39 bp of the *Mu10, 11,* and *12* TIRs have been sequenced [62]. However, the primer used to amplify the TIR ended in a 3′ GTC, allowing for the assumption that the sequence continues as GAC (shown as small case) and that the *Taq* ^α^I site remains intact. Of the most recently discovered *Mu* elements (13–19), only *Mu13* has been confirmed to actively move and create new mutations [58]. TIR sequences obtained from [3], [58], [62]–[68].

**Figure 2:**
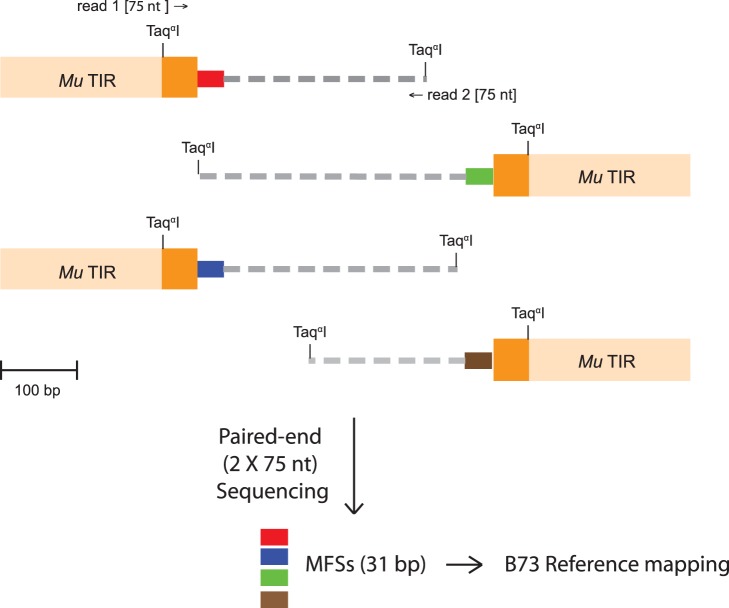
*Mu*-*Taq* ^α^I Library Construction. Digesting genomic DNA with *Taq* ^α^I creates a library of fragments. Fragments containing a *Mu*-MFS junction will all contain a degenerate 39 nt *Mu* TIR tag along with 31–35 nt of flanking genomic sequence at one end of the 2×75 nt paired-end read. These fragments are computationally identified and the *Mu* flanking sequence (MFS) is mapped to the maize reference genome.

To identify a suitable restriction enzyme, a ClustalW [41] alignment was performed on 70 bp of TIR sequences from all known active and potentially active (mobile) *Mu* elements (Figure 1). Of the enzyme options available in the *Mu* TIR (Table S4), *Taq* ^α^I and *Mnl*I fit the criteria we sought. *Taq* ^α^I was chosen because it recognizes a perfectly conserved 4 bp motif (TCGA), located 37–40 bp from the TIR end, and this enzyme was cytosine-methylation insensitive (NEB product information). Using this enzyme with barcoded adapters, we would generate enough data to identify a TIR tag of 39 bp and a MFS tag of 31 bp, ample enough sequence data to unambiguously identify *Mu* TIRs and to map MFS to the maize genome (Figure 2). [Note: using standard Illumina adapters, *Taq* ^α^I digests will generate a TIR sequence of 39 bp and a MFS sequence of 36 bp.].

### Identification of Mu Flanking Sequences

The basic strategy was to digest genomic DNA with *Taq* ^α^I, construct Illumina libraries, and pair-end sequence to identify MFS for mapping to the B73 reference genome (Figure 2). Genomic DNA samples were isolated from plants homozygous for several mutant alleles (Table S1) and digested using *Taq* ^α^I. The DNA fragments were prepared for Illumina sequencing by end-repair, adenylation, adapter ligation, and library amplification (see Materials and Methods). Each genomic library was ligated to a specific barcoded adapter for later identification. After qPCR normalization, the individual libraries were multiplexed and sequenced using the Illumina HiSeq 2000. The resultant sequencing data were demultiplexed and parsed by barcodes. Each parsed file was analyzed to identify reads containing a *Mu* TIR tag on one end, and its adjacent MFS extracted and parsed as fasta files. MFS were mapped to the B73 reference genome (version 2) using the BWA [34] and Bowtie 2 [42], [43].

The results of this analysis are summarized in Table 1. The average number of reads per sample was ∼14,500,000 reads, with a range from 8,955,215 to 25,196,240 reads. This range indicated that the qPCR step was fairly effective at normalizing the individual libraries in the multiplexed pool. Given that 150 bp of sequence are generated for each read, the average number of bases generated per sample was ∼2.15 Gb, which, if the reads were distributed evenly, is approximately 1X coverage of the entire maize genome [17]. Since we are only sampling a subset of the genome that is directly adjacent to a *Taq* ^α^I recognition site, however, the depth of coverage in these regions should be much higher.

**Table 1:** Summary of Sequenced Alleles.

Allele	Mu insertion^1^	Total Number ofPaired-End Reads	Number of MFSReads (total/unique)	Gene Identified(this study)
*a1-mum1*	Unknown^1^	21,746,017	506/163	No
*a1-mum2*	Yes	12,486,199	328/132	No
*a2-mum2*	Unknown^2^	11,122,765	230/120	No
*a2-mum4*	Unknown^2^	12,301,174	331/146	Yes
*bz1-mum4::Mu1*	Unknown^2^	9,824,747	263/167	No
*bz1-mum9*	Yes	9,100,676	228/101	Yes
*bz2-mVW4::MuDR*	Yes	8,955,215	224/120	Yes
*c2-mum1*	Unknown^2^	14,066,613	374/172	Yes
*wx1-mum1*	Unknown^2^	11,681,516	370/171	No
*wx1-mum2*	Unknown^3^	16,439,709	275/130	No
*wx1-mum5::Mu8*	Yes	18,280,957	217/100	Yes
*la1-mu1* (replicate 1)	Unknown	25,196,240	280/161	Yes
*la1-mu1* (replicate 2)	Unknown	10,754,601	144/105	Yes
*sk1-mu1*	Unknown	14,327,049	168/141	Yes

1 Allele shown to contain a *Mu* element by molecular analysis.

2 Uncharacterized at the molecular level but likely caused by *Mu* insertion based on mutable phenotype dependent on *Mutator* activity.

3 Atypical mutability pattern. May not contain *Mu* element.

**Figure 3:**
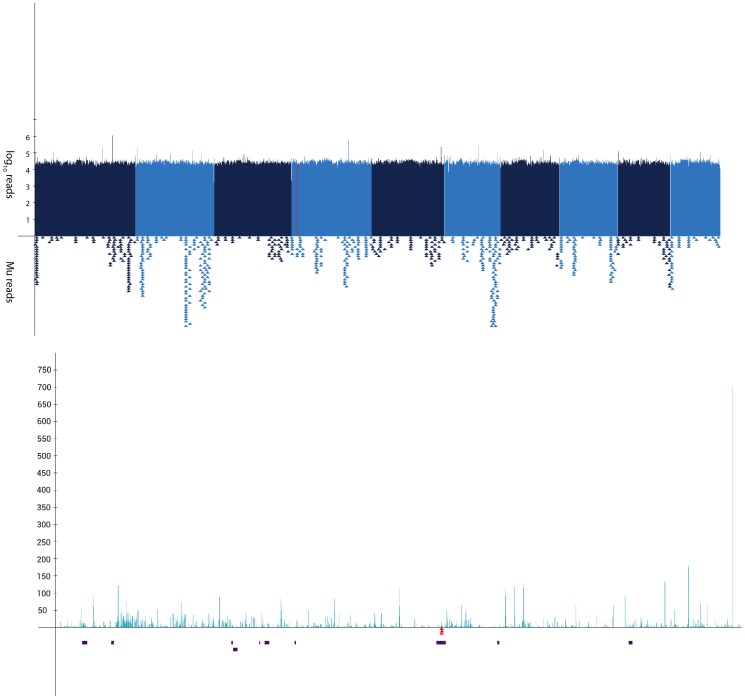
Manhattan plot of *la1-mu1 Taq* ^α^I sequencing. (**A**) Manhattan plot showing the distribution of reads from *la1-mu1* genomic DNA mapped throughout the B73 genome. Alternating colors represent each of the ten maize chromosomes. Each x-axis pixel represents a bin of 1 Mb and the logarithmic y-axis denotes the number of reads mapping to each bin. The red line represents the known genetic map position for the *la1* reference mutation. Each triangle below the plot represents the approximate location of mapped MFS. (**B**) Expanded Manhattan plot of a 1 Mb interval corresponding to the approximate map position of *la1*. Same as top with each x-axis pixel representing a bin of 1 kb. Filtered genes in the 1 Mb interval are shown as black rectangles. MFS mapping to this interval are shown as inverted red triangles.

To assess coverage, all paired-end reads from one library, *la1-mu1* were mapped to the maize B73 reference genome using the BWA algorithm [34]. These results showed a uniform distribution across the genome (Figure 3A). A closer look at the read coverage in a 1 MB subinterval (Figure 3B) showed a relatively even distribution of reads. Given that some *Taq* ^α^I fragments created through digestion of the genome will be either too small or too large to be sequenced and that some areas will not have a recognition site at all, it is not surprising that certain areas in the interval were not covered.

Next, the number of TIRs and associated MFS detected in each sample was analyzed. On average, 281, with a range of 144 to 506, were found in each genomic library (Table 1). Some of these MFS were likely independent reads from the same insertion, or the results of PCR replication. Because the paired end reads were fixed at restriction sites, it was not possible to distinguish between these two possibilities. Approximately 50% of the total MFS represent unique reads. Given that there were between 50 and 100 *Mu* elements per genome [reviewed in [10]] this number of unique TIRs (twice the number of expected insertions) was in the range expected for a genome containing active *Mu* elements. As long as there is not a strong bias towards amplifying *Mu* TIR fragments, PCR replicates should alter read depth evenly throughout the genome. If we were to assume the number of *Mu* TIRs was high (e.g. 200), the 39 bp *Mu* TIR tag that we search for would make up 0.000312% of the genome. Nonetheless, in our data, an average of 0.00204% of sequencing reads contained a *Mu* TIR and MFS, representing 6.5-fold enrichment for *Mu* TIR fragments.

### Analysis of Mutant Alleles

In total, we analyzed MFS from 13 different plant genomes. Eleven of the 13 genomes harbored a mutation in a known gene, while the remaining two genomes harbored mutations in previously unidentified genes, *lazy plant1* and *silkless1*. Each of these 13 mutations was derived from *Mutator* lines of maize (see Table S1 for alleles and sources) and four of these were previously known to be caused by an insertion of a *Mu* element. The molecular basis of the remaining nine alleles had not been previously characterized at the molecular level. Seven of these nine alleles showed mutability in the presence of *Mutator* activity, a likely indicator of Mu insertion in the gene.

The locations of MFS found in each of these genomes were examined for those mapping to the genomic location of the affected gene. In the case of the four known *Mu*-induced mutations, our analysis identified *Mu* insertions in three of these four genes. In the previously uncharacterized alleles, we identified a *Mu* insertion in four out of nine alleles. These included the two previously uncharacterized genes, *lazy plant1* (discussed below) and *silkless1* (manuscript in preparation) both derived from active *Mutator* stocks.

**Figure 4:**
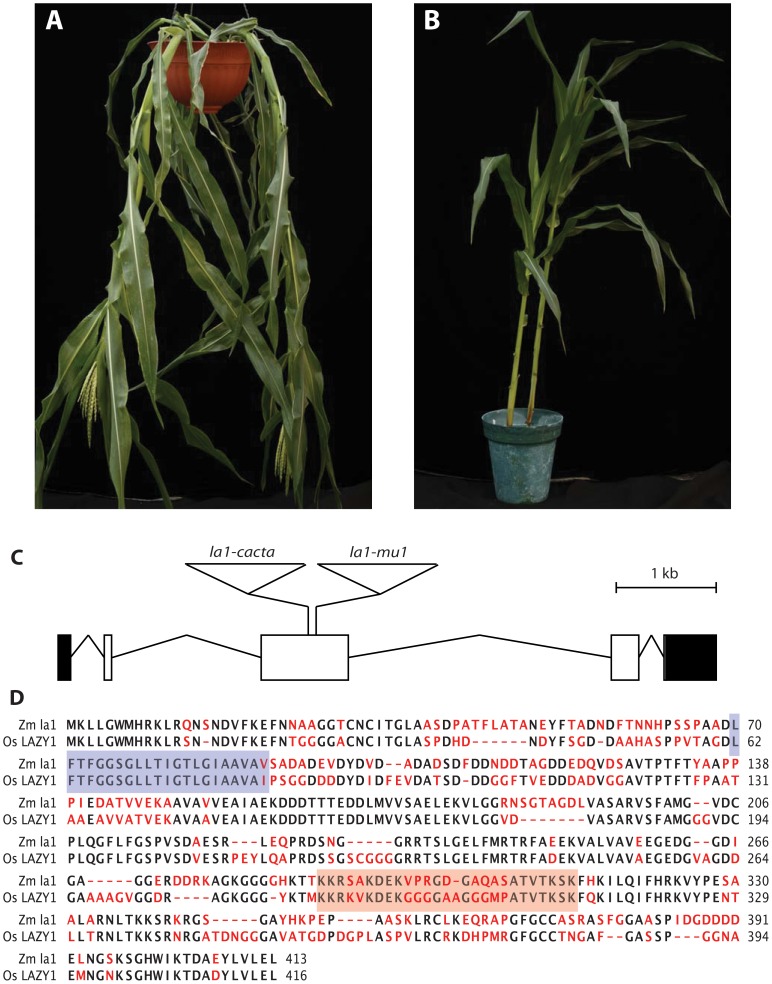
Identification of *la1*. The *la1* mutant plants (**A**) exhibit prostrate growth habit caused by a lack of a negative gravitropic response. Wild type maize plants (**B**) purposefully grow away from gravity (negative gravitropism). (**C**) Gene structure of *la1* with the site of the *Mu* insertion in the *la1*-*mu1* mutant allele and the CACTA family insertion in the *la1-cacta* allele. Exons are shown as empty boxes and UTRs are shown as filled boxes. The *Mu* insertion has not been characterized and is not to scale. (**D**) Amino acid ClustalW alignment of the maize and rice predicted LAZY1 proteins. The pair shares 60% identity. The predicted transmembrane domain is shaded in blue, the predicted NLS domain is shaded in orange.

The *lazy plant1* (*la1*) maize mutation results in a prostrate growth pattern (Figure 4A). Interestingly, mutant plants do not have any type of structural abnormality but instead lack the drive to grow upwards, leading to their appropriately named “lazy” phenotype [44]–[46]. Charles Darwin was one of the first scientists to document that plant shoots show negative gravitropism, *i.e.* grow in the opposite direction as gravity [47]. The *lazy plant1* mutants have been shown to continue growing in whichever direction they are pointed in, regardless of the direction of gravity [44]. Without the perception of gravity, the maturing plant grows prostrate (Figure 4A). These phenotypes suggest that the wild type gene is required for the process of negative gravitropism in the shoot.

The *la1* gene of maize had not been cloned at the time of this study but its chromosomal position had been genetically mapped to chromosome 4 (Maize IBM2 2008 Neighbors map, www.maizegdb.org). In 2009, a recessive “lazy” mutation was found segregating in an F2 population derived from an active Robertson’s *Mutator* line. This mutation represented an ideal situation for testing our method since any MFS in a gene mapping to the genetic location of *la1* in these plants would potentially represent a candidate *La1* gene. The Mu-Taq strategy was applied to two biological replicates of *la1* plants. The libraries returned 25,196,240 reads with 280 MFS and 10,754,601 reads with 144 MFS, respectively (shown diagrammatically in Figure 3A). In both libraries, a single MFS mapped within a few MB of the predicted *la1* map location (Figure 3B). This MFS, identical in both replicates, mapped to a predicted exon within the GRMZM2G135019 gene (*la1-mu1;* Figure 4C) located on chromosome 4 at position 17,977,557 to 17,984,094, an estimated 4 Mb from the genetic map position of *la1.* BLASTn analysis of the predicted cDNA sequence returned no putative conserved domains but did identify an *Oryza sativa* (rice) gene (Os11g0490600) with significant homology (*E* value = 8e^−68^). In rice, mutant alleles of Os11g0490600 display a gravitropic phenotype similar to the maize “lazy” phenotype [48]. Moreover, the maize and rice genes are located in a region of extensive synteny [49] suggesting that they are orthologous. BLASTp [50] alignment of the maize and rice proteins indicates that they share 60% identity and 67% similarity (Figure 4D). These results indicate that GRMZM2G135019, identified by virtue of its association with a linked MFS found through Mu*-*Taq analysis, is a strong candidate for the *lazy plant1* gene of maize.

Confirmation of the identification of the *La1* gene was obtained by characterizing an independent *la1* mutation. Primers derived from the GRMZM2G135019 gene were used to amplify and sequence an independent *la1* mutant alleles obtained from the Maize Stock Center. Sequencing of this allele revealed disruption of GRMZM2G135019 by the insertion of a previously uncharacterized TIR family CACTA transposable element (*la1-cacta*; Figure 4C). Complementation test of this allele with the *la1-reference* allele (Maize Genetics Cooperative Stock Center) provide further confirmation that GRMZM2G135019 is indeed the *la1* gene.

### Characterization of the Maize *La1* Gene

**Figure 5:**
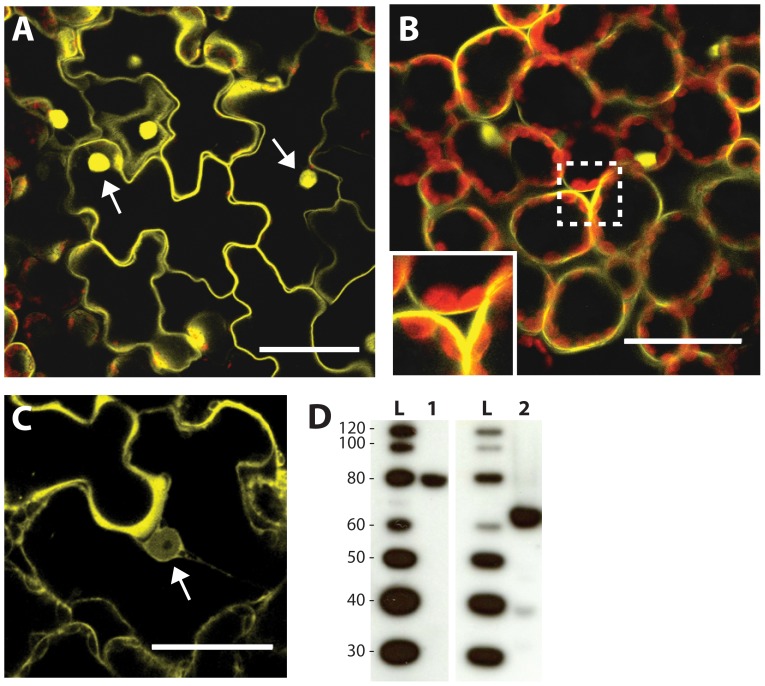
Localization of LA1. LA1-Citrine localizes to the nucleus (arrows) and cell membrane in (A) *N. benthamiana* epidermal cells and (B) *N. benthamiana* mesophyll cells. Inset shows detail of the mesophyll cell membrane. (C) Deletion of the putative NLS in LA1ΔNLS-Citrine abrogates nuclear localization. Scale bars are 20 uM. (D) Western blotting confirms expression of full length LA1-Citrine (lane 1) and LA1ΔNLS-Citrine (lane 2).

The maize *La1* was predicted to encode a protein of 413 amino acids of unknown function with a molecular weight of 43.6 kD (Figure 4D). Prediction of transmembrane helices using both HMMTOP [51], [52] and TMpred [53] indicated a strong likelihood of the presence of a transmembrane helix at position 70–91 (blue box in Figure 4D). Strong homology was also seen at an NLS region previously described in rice *LAZY1* at position 288–313 (orange box in Figure 4D). The subcellular localization of maize LA1 was determined with a fluorescent-tagged LA1 protein (*La1*:Citrine). LA1 localized to the cell membrane and nucleus during transient expression in *N. benthamiana* epidermal and mesophyll cells (Figure 5A, 5B). The LA1-Citrine fusion protein was predicted to be larger than the threshold for cytoplasm-to-nucleus diffusion through the nuclear pores (< ∼50 KDa), and LA1-Citrine was not detected in the cytoplasm, suggesting that nonspecific nuclear localization was unlikely. Western blotting also revealed no evidence of smaller cleavage products containing citrine that could cause artifactual nuclear signal (Figure 5D). To confirm that the putative maize LA1 NLS domain was required for nuclear localization, a deletion encompassing the NLS (amino-acid residues 263–389) was generated (*La1*ΔNLS:Citrine). Deletion of the NLS in LA1ΔNLS-Citrine abrogated nuclear localization (Figure 5C). These results suggest that full-length LA1 protein is present at both the cell membrane and nucleus, though its molecular function remains unclear.

## Discussion

The Mu-Taq method identified three out of four alleles known to be caused by *Mu* insertions and four out of nine alleles (including *la1* and *sk1*) that were potentially caused by insertion of a *Mu* element. This efficiency of known alleles (75%) is comparable to that of previously published methods such as DLA (86%) and the biotinylated oligonucleotide method (80%). The overall efficiency of the method, however, was lower for previously uncharacterized alleles. For example, it is likely that many of these unknown alleles actually contain *Mu* insertions since their mutable phenotype is dependent on *Mutator* activity. This would therefore place a lower limit on the efficiency at 54%. From analysis of bulk read data, we detect over 100 unique MFS in a plant known to harbor *Mu* elements (Table 1). This is above the number detected per genome by other methods and in the range predicted from the average number of *Mu* elements in a genome. Given that the *a1-mum2* allele is confirmed to have a *Mu* insertion [54] and that other alleles showing mutability are likely to be *Mu*-induced, certainly a fraction of MFS have gone undetected. Based on the number of unique MFS identified in each genome the depth of sequencing is probably below saturation and additional sequencing may lead to the identification of some of these alleles. Therefore, the efficiency of our method at detecting a *Mu* insertion is between the estimates of 54–75% [54].

Besides depth of sequencing, there are many possible reasons for not detecting a MFS when it is present in the genome. Some of these reasons are technical in nature. One obvious reason would be the absence of the conserved *Taq* ^α^I site in the TIR. Based on the conservation of *Taq* ^α^I in all known mobile and potentially mobile elements, we believe that this possibility is unlikely. Another reason is that any individual *Taq* ^α^I fragment generated during restriction digestion may be too large for efficient cluster formation, estimated to be greater than 800 bp. While the average size of a *Taq* ^α^I fragment, based on a random distribution of bases and an AT-GC content of 50%, would be 256 bp, the actual size will vary according to local and global base composition and a quasi-random distribution of sites. Also, given that 130 bp of adapter sequence are added during library construction, it is possible that some *Taq* ^α^I fragments will exceed the size necessary to generate efficient clusters during the sequencing process. Nevertheless, this is predicted to be a relatively minor fraction of *Taq* ^α^I fragments. A third possible reason for missing an individual MFS is the problem of *Taq* ^α^I digesting too close to the end of the TIR sequence to identify a MFS. Since we are mapping MFS using the adjacent 31 nt, the frequency of smaller size fragments is not expected to be significant. In the specific case of *Mu* elements and *Taq* ^α^I, the terminal two bases of the TIR are part of the *Taq* ^α^I recognition site. Therefore, on average, one out of sixteen TIR ends would reconstitute the full *Taq* ^α^I site and be cut directly after the TIR. These fragments would have no detectable MFS.

It is also possible that the map position of an individual MFS is not accurate due to mapping errors or reference genome limitations. Our current analysis includes only the single, most significant mapped hit. Given that variation exists between the maize lines used in this study and the inbred B73 reference genome as well as the relatively high Illumina sequencing error rate, some MFS may not have been properly mapped. In the short term, reducing the stringency of possible matches and mapping both ends of the paired-end reads may resolve this issue. A longer-term solution would be to continue to characterize and catalog the variation found in maize lines. Nevertheless, for these issues to be relevant, both ends of the transposon would have to suffer from one or more of these problems to cause the element to be completely absent from a particular MFS dataset.

Another issue with the efficiency of MFS detection is depth of read coverage and absence of coverage for some areas of the genome. As previously mentioned in cases where the gene went undetected in the MFS reads, it is possible that read depth was insufficient to detect all MFS sequences. In our experiments we achieve an overall depth of coverage of approximately 1X. However, since only fragments adjacent to a *Taq* ^α^I sites were sequenced, our effective genome size is considerably smaller and our effective depth of coverage much higher for these regions. Our estimate of 6.5X enrichment for *Mu* TIR fragments should enhance the effectiveness of this method to detect individual MFS. Evidence that depth of coverage may not be a significant issue is that the allele with the lowest number of total paired-end reads (*bz2-mVW4*) was detected, but the allele with the second highest number of total paired-end reads (*a1-mum1*) was not. Therefore, increasing read depth may not be the only solution sought to resolve the issue of MFS detection.

One biological reason for failing to identify an MFS-associated gene is that the mutant allele may not contain a *Mu* element. Since *Mu* is mobile, it may no longer be associated with a particular allele. *Mu* elements frequently insert and excise imperfectly, causing loss-of-function alleles even though the *Mu* element is no longer present. In our study, nine alleles derived from *Mutator* lines were not previously characterized at the molecular level to confirm *Mu* insertions in the gene. Nevertheless, the mutable phenotype associated with many of these alleles makes it likely that a Mu insertion is the causative agent. Yet, only four of these nine alleles were found to be associated with a particular MFS. It has been well documented that many mutations recovered from *Mu* lines do not contain heritable *Mu* insertions. For instance, at the *liguleless1* locus of maize, two alleles derived from *Mutator* stocks did not contain *Mu* insertions [55], while at the *tasselseed1* locus, only two out of four alleles derived from *Mutator* lines contained *Mu* insertions [56]. If the alleles used in this study did not contain a *Mu* element, this would explain the failure to detect at least some of these genes in the dataset. At this point, the molecular basis of the mutations in the alleles that were not identified need to be further investigated to determine whether or not they actually contain *Mu* elements with conserved TIR sequences. This additional data would allow us to accurately assess the effectiveness of our protocol.

### Comparison to Other Methods

Three other methods have been reported to massively identify MFS using next-generation sequencing. Though successful, these methods rely on imperfectly conserved sequences in the *Mu* TIR (Figure 1). The DLA method [30] uses a set of degenerate primers to amplify out of the *Mu* TIR to create a library of MFS. Certain *Mu* elements are likely to be selectively amplified because of differential annealing efficiency of the various primers. The biotinylated oligonucleotide method [31] relies on annealing over a larger range of the TIR, expanding the possibility that divergent sequences of certain *Mu* elements limit their capture and sequencing. A recent method called Mu-seq incorporates a single 23 base, 12-fold-degenerate *Mu* TIR primer to target fragment amplification [57]. In comparison, our method did not rely on degenerate *Mu* primers but rather on anchoring reads to a highly conserved of a 4 bp *Taq* ^α^I site, which is located in the MURA transposase binding site. This site has been perfectly conserved in all active *Mu* elements characterized to date. In addition, our method would identify MFS from any unknown *Mu* elements as they likely contain the conserved TIR *Taq* ^α^I site. Seven new *Mu* elements were recently identified [58] and it seems likely that other *Mu* elements have yet to be identified. In our method, the identification of TIR tags was incorporated into an algorithm that could be easily adapted to include additional TIR elements and to modulate the stringency and flexibility of TIR identification.

One of the main advantages of the Mu-Taq strategy is its simplicity. Standard Illumina reagents and adapters can be used for libraries and sequencing with little protocol modification. Other published methods are cumbersome and require numerous steps to prepare sequencing libraries or complicated post-library enrichment methods. For instance, the DLA method requires a dideoxynucleotide blocking step as well as an extra PCR amplification, introducing biases and read redundancy into the libraries. The biotinylated oligonucleotide method requires two rounds of hybridization as well as an extra amplification, also introducing amplification bias and read redundancy. Mu-seq employs three rounds of PCR amplification and special adapters. In contrast, our Mu-Taq method requires only the typical steps – end-repair, adenylation, and ligation – used during standard Illumina library construction. Next-generation library protocols also require a size fractionation step. In this method, instead of the standard random DNA fragmentation by sonication or nebulization, a simple restriction digestion step was employed as both a size fractionation and enrichment step. Once end-repaired and adenylated, these fragments can be ligated to standard Illumina adapters and amplified in a single low cycle PCR reaction. Subsequently, the Mu-Taq method incorporates a custom bioinformatics pipeline to identify *Mu* TIR sequences to extract MFS and standard algorithms to map their corresponding MFS.

While other NGS methods generate a much higher percentage of reads that correspond to MFS, their exceptional coverage depth is unnecessary for some applications such as single gene identification. Even though in our method only 0.00204% of reads correspond to MFS, mutations in eight independent plant genomes were identified from reads derived from a single Illumina HiSeq lane. With the ever-improving efficiency of NGS platforms, an increase in read depth will become even more superfluous for regular gene cloning applications. While larger MFS screening projects may wish to identify more alleles in a single run using an MFS amplification protocol, our method is ideal for identifying single or multiple *Mu*-tagged genes of interest.

### Adaptability of Mu-Taq to Other Transposon Families

The Mu-Taq method is readily adaptable to other transposon systems, and indeed any type of known DNA insertion, in organisms besides plants. The only requirement is the presence of a suitable and highly conserved restriction site close to the end of the insertion. The “Dynamic TIR Finder” algorithm has been written to accept a Fasta file of any set of TIR tags and to parse out all corresponding transposon flanking sequence reads. Therefore, in principle, this method is readily adaptable to any species, and to any family of transposable elements with conserved terminal elements. In addition, the method should be readily adaptable to other sequencing platforms. In this way, the Mu-Taq method described here represents a general and flexible strategy for identifying transposon-tagged genes in the ever-progressing NGS field.

### Application of Mu-Taq Cloning to Identify and Characterize Maize *la1*

The application of the Mu-Taq method effectively identified the maize *la1* gravitropism gene in two biological replicates of an allele derived from a *Mutator* line. We were able to further identify a second mutant *la1* allele that failed to complement the *la1-reference* that contained a previously uncharacterized CACTA transposon insertion. We show that maize *La1* shares significant homology to its recently identified rice ortholog [48]. Concurrent with this study, Dong et al. (2013) have also reported on the cloning of the maize *La1* gene using a Mutator-derived allele [59]. Interestingly, the mapping and cloning strategy employed required a time- and labor-intensive effort involving several generations and nearly 1300 plants. In our study, from mutant to candidate gene identification required only a few weeks at a substantial savings in time and expense.

Both our study and Dong et al. [59] show that GRMZM2G135019 corresponds to the maize *La1* gene and LA1 protein localizes to the nucleus and cell membrane, a localization profile previously reported for both the rice and *Arabidopsis La1* orthologs [48], [60]. Using sequence homology between rice and maize *La1*, we confirm a putative NLS required for nuclear localization of maize LA1. The rice *LAZY1* gene has been shown to affect gravitropism through asymmetric auxin distribution in the developing plant [48], [61]. Similar perturbations of auxin transport have been reported in maize *la1* plants [59], and the *la1* phenotype was shown to be auxin dependent in the original investigations into the nature of the mutant in the 1930s [45]. Even though the effector (auxin) is known, there still remain significant questions regarding how *la1* actually senses gravity. Although trafficking of membrane-bound LA1 to the nucleus could hypothetically mediate auxin signaling, we are unable to detect shuttling of LA1 by confocal microscopy. Furthermore, a recent study showed that deletion of the AtLAZY1 NLS did not prevent rescue of the *atlazy1* mutant phenotype in Arabidopsis, leaving the functional relevance of nuclear LA1 unclear [60]. More complete understanding of LA1 activity will certainly be a productive area of future research on gravitropism.

## Supporting Information

Table S1 **Mutant alleles used in this study.**
(DOCX)

Table S2 **Barcoded adapters used in this study.**
(DOCX)

Table S3 **Primers used in this study.**
(DOCX)

Table S4 **Restriction enzyme choices in the first 70 bp of** ***Mu*** **TIRs.**
(DOCX)

File S1 **MuTaq Supplementals.**
(ZIP)
