# Supplementary material for: Identification of the Maize Gravitropism Gene lazy plant1 by a Transposon-Tagging Genome Resequencing Strategy
Source: PLoS One. 2014 Jan 31;9(1):e87053. doi: 10.1371/journal.pone.0087053 (PMC3909067; doi:10.1371/journal.pone.0087053)
Supplement: Table S1 — Mutant alleles used in this study. (DOCX) [file pone.0087053.s001.docx]

**Table S1: Mutant alleles used in this study**

| **Allele** | **Mu insertion** | **Source** |
| --- | --- | --- |
| *a1-mum1* | Unknown^[[1]](#footnote-1)^ | D. Robertson |
| *a1-mum2* | Yes | MGCSC^[[2]](#footnote-2)^ |
| *a2-mum2* | Unknown | D. Robertson |
| *a2-mum4* | Unknown | D. Robertson |
| *bz1-mum4::Mu1* | Unknown | MGCSC |
| *bz1-mum9* | Yes | M. Freeling in Robertson *Mu* line |
| *bz2-mVW4::MuDR* | Yes | V. Walbot |
| *c2-mum1* | Unknown | D. Robertson |
| *la1-cacta^[[3]](#footnote-3)^* | No | MGCSC |
| *la1-mu1* | Yes | J. Mottinger, this study |
| *la1-reference* | No | MGCSC |
| *sk1-mu* | Yes | J. Mottinger, this study |
| *wx1-mum1* | Unknown | MGCSC |
| *wx1-mum2* | Unknown | MGCSC |
| *wx1-mum5::Mu8* | Yes | MGCSC |

1. Alleles with suffix designation “*-mum*” show mutability in the presence of *Mutator*  activity and likely caused by insertion of  *Mu* element. [↑](#footnote-ref-1)
2. Maize Genetics Cooperation Stock Center [↑](#footnote-ref-2)
3. Complementation tests with *la1-reference* allele performed by MGCSC (personal communication) [↑](#footnote-ref-3)
