# Supplementary material for: Identification of the Maize Gravitropism Gene lazy plant1 by a Transposon-Tagging Genome Resequencing Strategy
Source: PLoS One. 2014 Jan 31;9(1):e87053. doi: 10.1371/journal.pone.0087053 (PMC3909067; doi:10.1371/journal.pone.0087053)
Supplement: Table S2 — Barcoded adapters used in this study. (DOCX) [file pone.0087053.s002.docx]

**Table S2:** **Barcoded adapters used in this study**

| *Name* | *Sequence (****barcode*** *and* *3’ T overhang)* *(5’ to 3’)* | *Library* |
| --- | --- | --- |
| NBC4F_01 | ACACTCTTTCCCTACACGACGCTCTTCCGATCT**ATGC**T | *bz2-mVW4::MuDR* |
| NBC4R_01 | [Phos]**GCAT**AGATCGGAAGAGCGGTTCAGCAGGAATGCCGAG |  |
| NBC4F_02 | ACACTCTTTCCCTACACGACGCTCTTCCGATCT**AAAA**T | *a2-mum4* |
| NBC4R_02 | [Phos]**TTTT**AGATCGGAAGAGCGGTTCAGCAGGAATGCCGAG |  |
| NBC4F_03 | ACACTCTTTCCCTACACGACGCTCTTCCGATCT**AATT**T | *la1-mum* (replicate 1) |
| NBC4R_03 | [Phos]**AATT**AGATCGGAAGAGCGGTTCAGCAGGAATGCCGAG |  |
| NBC4F_10 | ACACTCTTTCCCTACACGACGCTCTTCCGATCT**AGTC**T | *bz1-mum9* |
| NBC4R_10 | [Phos]**GACT**AGATCGGAAGAGCGGTTCAGCAGGAATGCCGAG |  |
| NBC4F_11 | ACACTCTTTCCCTACACGACGCTCTTCCGATCT**AGGA**T | *la1-mum* (replicate 2) |
| NBC4R_11 | [Phos]**TCCT**AGATCGGAAGAGCGGTTCAGCAGGAATGCCGAG |  |
| NBC4F_17 | ACACTCTTTCCCTACACGACGCTCTTCCGATCT**TAAT**T | *a1-mum1* |
| NBC4R_17 | [Phos]**ATTA**AGATCGGAAGAGCGGTTCAGCAGGAATGCCGAG |  |
| NBC4F_18 | ACACTCTTTCCCTACACGACGCTCTTCCGATCT**TATA**T | *bz1-mum4::Mu1* |
| NBC4R_18 | [Phos]**TATA**AGATCGGAAGAGCGGTTCAGCAGGAATGCCGAG |  |
| NBC4F_25 | ACACTCTTTCCCTACACGACGCTCTTCCGATCT**TGAC**T | *a1-mum2* |
| NBC4R_25 | [Phos]**GTCA**AGATCGGAAGAGCGGTTCAGCAGGAATGCCGAG |  |
| NBC4F_26 | ACACTCTTTCCCTACACGACGCTCTTCCGATCT**TGTG**T | *wx1-mum1* |
| NBC4R_26 | [Phos]**CACA**AGATCGGAAGAGCGGTTCAGCAGGAATGCCGAG |  |
| NBC4F_33 | ACACTCTTTCCCTACACGACGCTCTTCCGATCT**GAAG**T | *c2-mum1* |
| NBC4R_33 | [Phos]**CTTC**AGATCGGAAGAGCGGTTCAGCAGGAATGCCGAG |  |
| NBC4F_34 | ACACTCTTTCCCTACACGACGCTCTTCCGATCT**GATC**T | *wx1-mum2* |
| NBC4R_34 | [Phos]**GATC**AGATCGGAAGAGCGGTTCAGCAGGAATGCCGAG |  |
| NBC4F_41 | ACACTCTTTCCCTACACGACGCTCTTCCGATCT**GGAA**T | *a2-mum2* |
| NBC4R_41 | [Phos]**TTCC**AGATCGGAAGAGCGGTTCAGCAGGAATGCCGAG |  |
| NBC4F_42 | ACACTCTTTCCCTACACGACGCTCTTCCGATCT**GGTT**T | *wx1-mum5::Mu8* |
| NBC4R_42 | [Phos]**AACC**AGATCGGAAGAGCGGTTCAGCAGGAATGCCGAG |  |
