# Supplementary material for: Identification of the Maize Gravitropism Gene lazy plant1 by a Transposon-Tagging Genome Resequencing Strategy
Source: PLoS One. 2014 Jan 31;9(1):e87053. doi: 10.1371/journal.pone.0087053 (PMC3909067; doi:10.1371/journal.pone.0087053)
Supplement: Table S3 — Primers used in this study. (DOCX) [file pone.0087053.s003.docx]

**Table S3: Primers used in this study**

| **Name** | **Sequence (5’ to 3’)^^[[1]](#footnote-1)^^** | **Purpose** |
| --- | --- | --- |
| PEMAM01 | *AATGATACGGCGACCACCGAGAT*CT**ACACTCTTTCCCTACACGA** | Illumina library amplification |
| PEMAM02 | *CAAGCAGAAGACGGCATACGA*GATCGGT**CTCGGCATTCCTGCTGAAC** | Illumina library amplification |
| P4619 | *AATGATACGGCGACCACCGAGAT* | qPCR for normalization |
| P4620 | *CAAGCAGAAGACGGCATACGA* | qPCR for normalization |

1. Bolded letters for the Illumina primers indicate the bases that will anneal to the adapters. The qPCR primers will only anneal to fragments that have the Illumina primers on either end (shown in italics). [↑](#footnote-ref-1)
