# Supplementary material for: Identification of the Maize Gravitropism Gene lazy plant1 by a Transposon-Tagging Genome Resequencing Strategy
Source: PLoS One. 2014 Jan 31;9(1):e87053. doi: 10.1371/journal.pone.0087053 (PMC3909067; doi:10.1371/journal.pone.0087053)
Supplement: Table S4 — Restriction enzyme choices in the first 70 bp of Mu TIRs. (DOCX) [file pone.0087053.s004.docx]

**Table S4: Restriction enzyme choices in the first 70 bp of *Mu* TIRs**

| **Name** | **Recognition site**  **(5’ to 3’)** | **Cut site distance from *Mu* end (bp)** | **Average cut frequency**  **(1 in _ bp)** | **CpG**  **Methylation Sensitivity^^[[1]](#footnote-1)^^** | **Percentage of *Mu* TIRs with intact site^^[[2]](#footnote-2)^^** |
| --- | --- | --- | --- | --- | --- |
| *Tsp*509I | AATT | 5 | 256 | No | 97% |
| *Fai*I | YATR | 17 | 64 | unknown | 100% |
| *Ear*I | CTCTTC | 18 | 4,096 | Yes | 72% |
| *Hpy*AV | CCTTC | 24 | 1,024 | Yes | 41% |
| *Mbo*II | GAAGA | 32 | 1,024 | No | 72% |
| *Tfi*I | GAWTC | 36 | 512 | Yes | 72% |
| *Hin*fI | GANTC | 36 | 256 | Yes | 72% |
| *Taq*^α^I | TCGA | 39 | 256 | No | 100% |
| *Mnl*I | CCTC | 43 | 256 | No | 100% |
| *Hpy*99I | CGWCG | 44 | 512 | Yes | 97% |
| *Cvi*JI | RGCY | 65 | 64 | No | 97% |

1. Plant DNA contains cytosine methylation at both CpG and CNG sites [67,68]. The sensitivity of most restriction enzymes to the latter form of cytosine methylation is not often known. [↑](#footnote-ref-1)
2. Calculated based on 32 TIRs for the first 40 bp and 26 TIRs for the last 30 bp (see Figure 1) [↑](#footnote-ref-2)
